# Serum proteomics in giant cell arteritis in response to a three-day pulse of glucocorticoid followed by tocilizumab monotherapy (the GUSTO trial)

**DOI:** 10.3389/fimmu.2023.1165758

**Published:** 2023-05-23

**Authors:** Lisa Christ, Andrea D. Gloor, Florian Kollert, Timo Gaber, Frank Buttgereit, Stephan Reichenbach, Peter M. Villiger

**Affiliations:** ^1^ Department of Rheumatology and Immunology, Inselspital, Bern University Hospital, University of Bern, Bern, Switzerland; ^2^ Department of General Internal Medicine, Inselspital, Bern University Hospital, University of Bern, Bern, Switzerland; ^3^ Department of Rheumatology and Clinical Immunology, Charité - Universitätsmedizin Berlin, Corporate Member of Freie Universität Berlin and Humboldt Universität zu Berlin, Berlin, Germany; ^4^ University of Bern, Institute for Social and Preventive Medicine, Bern, Switzerland; ^5^ Medical Center Monbijou, Rheumatology and Immunology, Bern, Switzerland

**Keywords:** giant cell arteritis, tocilizumab, glucocorticoid, proteomics, biomarker, disease activity

## Abstract

**Objective:**

Proteome analyses in patients with newly diagnosed, untreated giant cell arteritis (GCA) have not been reported previously, nor are changes of protein expression upon treatment with glucocorticoids (GC) and/or tocilizumab (TCZ) known. The GUSTO trial allows to address these questions, provides the opportunity to learn about the differential effects of GC and TCZ on proteomics and may help to identify serum proteins to monitor disease activity.

**Methods:**

Serum samples obtained from 16 patients with new-onset GCA at different time points (day 0, 3, 10, and week 4, 24, 52) during the GUSTO trial (NCT03745586) were examined for 1436 differentially expressed proteins (DEPs) based on proximity extension assay technology. The patients received 500 mg methylprednisolone intravenously for 3 consecutive days followed by TCZ monotherapy.

**Results:**

When comparing day 0 (before the first GC infusion) with week 52 (lasting remission), 434 DEPs (213↑, 221↓) were identified. In response to treatment, the majority of changes occurred within 10 days. GC inversely regulated 25 proteins compared to remission. No difference was observed between weeks 24 and 52 during established remission and ongoing TCZ treatment. Expression of CCL7, MMP12, and CXCL9 was not regulated by IL6.

**Conclusion:**

Disease-regulated serum proteins improved within 10 days and were normalized within 24 weeks, showing a kinetic corresponding to the gradual achievement of clinical remission. The proteins inversely regulated by GC and TCZ shed light on the differential effects of the two drugs. CCL7, CXCL9, and MMP12 are biomarkers that reflect disease activity despite normalized C-reactive protein levels.

## Introduction

Giant cell arteritis (GCA) is the most common systemic vasculitis in elderly adults. It is characterized by invading of immune cells through the adventitial vasa vasorum network in the immune-privileged three-layered vessel wall (1). Upon activation of T cells, activated macrophages and T cells form granulomatous lesions and release a variety of chemokines, inflammatory cytokines, growth factors, and enzymes. Chemokines such as CCL19 and CCL21 attract other immune cells, whereas cytokines like interferon-gamma (IFNγ), tumor necrosis factor-alpha (TNFα), interleukin (IL)1b, IL2, IL6, IL17, IL21, and IL22 are responsible for a local and systemic inflammatory response. Growth and proangiogenic factors including platelet-derived growth factor (PDGF) and vascular endothelial growth factor (VEGF) lead to lumen-narrowing intimal hyperplasia with neovascularization. Enzymes such as matrix metalloprotease (MMP)-9 cause degeneration of the extracellular matrix, which facilitates further immigration of immune cells and leads to weakening of the vessel wall and formation of aneurysms (1).

The current definition of disease activity in GCA is based on symptoms, findings in imaging, as well as the inflammatory markers C-reactive protein (CRP) and erythrocyte sedimentation rate (ESR) (2). Tocilizumab (TCZ), a monoclonal antibody targeting the IL6 receptor (IL6R), has proven efficacy in GCA treatment, but inhibits the hepatic acute phase response. Hence, CRP and ESR are unreliable for monitoring disease activity in TCZ-treated patients (3, 4).

Until now, most studies on serum proteins in GCA were hypothesis-driven and used pre-specified panels of proteins, e.g. cellular markers, cytokines and effector molecules. Several proteins, like ANGPT2, CD163, CHI3L1, CXCL13, CXCL9, CXCL10, IL1B, IL18, IL2RA, IL6, IL6R, MMP2, MMP3, PTX3, S100A9, SPP1, TEK, TIMP1, TNC, TNFRSF1A, and VEGFA, were described as potential markers of active GCA. Furthermore, most data were generated using sera of patients pre-treated with glucocorticoids (GC) (5–12). This precluded differentiation between changes induced by disease versus changes induced by GCs.

A multiplex analysis bears the advantage of identifying previously unrecognized molecules, while offering the opportunity to confirm or reject hypotheses.

The goals of our study were (i) to describe the profile of serum proteins in active, newly diagnosed naïve GCA patients compared to patients in lasting remission at week 52, (ii) to investigate and compare the effects of pulsed GC treatment and ensuing long-term TCZ monotherapy on changes in serum protein expression pattern, and (iii) to identify serum proteins which are not under the control of IL6 and, thus, may qualify as biomarkers of disease activity in patients with GCA, especially in patients treated with TCZ.

## Patients and methods

### Patients and samples

Patients with newly diagnosed GCA were enrolled in the GCA treatment with ultra-short GCs and TCZ (GUSTO) trial and received 500 mg/d methylprednisolone intravenously for 3 consecutive days (NCT03745586) (13). Thereafter, GC treatment was discontinued, and a single dose of TCZ (8 mg/kg body weight) was administered intravenously, followed by weekly subcutaneous TCZ injections (162 mg) from day 10 until week 52. Serum samples were collected according to standard operating procedures (processed 30 minutes after venipuncture, centrifugation at 2730 r.p.m. for 10 min) prior to treatment or with the least prior GC exposure (referred to as day 0), at day 3 (after GC treatment), and during TCZ monotherapy at day 10 and weeks 4, 24, and 52 (**Supplementary Figure 1**). Samples were stored at −80°C until further analysis.

A total of 18 patients were included in the GUSTO study and 13/18 patients completed the 52-week treatment course. 16/18 patients contributed samples for this study. In 13/16 patients, the first sample was obtained prior to any GC treatment, 3/16 patients had a median of 3 days (1, 3, and 7 days respectively) of prior oral GC treatment (daily dose: 20-60 mg prednisolone). Samples from all 13 patients completing the study were analyzed (6 time points/patient). Additionally, two samples (day 0, week 4) from one patient who discontinued the study due to diverticulitis after achieving remission, and six samples (day 0, 3, and 10, each) from two patients who discontinued the study after day 10 due to persistent cranial symptoms were included. In total, 86 samples were assessed. Baseline characteristics are displayed in the **Supplementary Table 1**.

### Measurements and statistics

Serum samples were analyzed on Olink Explore 1536 (Uppsala, Sweden), a proximity extension assays (PEA) multiplex immunoassay platform capturing 1463 proteins (14).

Normalized protein expression values (NPX) were used for analysis. Principal component analysis (PCA) and sample-wise NPX-distribution was evaluated for outliers. Samples identified as outliers were excluded from downstream analysis along with those with quality control (QC)-warning status.

Protein-wise t-tests compared NPX-values between the two groups (threshold, adjusted p-value < 0.05) and results were adjusted for multiple testing (Benjamini-Hochberg) to identify differentially expressed proteins (DEPs). In the first step, we investigated whether there was a difference in day 0 samples of those patients with as compared to without prior GC treatment. In case of a difference due to previous GC treatment, we intended to analyze both groups separately. In a second step, we compared samples between day 0 versus day 3 (referred to as GC treatment), week 4 versus week 52 (referred to as TCZ treatment), day 0 versus week 52 (referred to as remission), day 3 versus week 4 (weaning effect of GC and evolving effect of TCZ treatment), and week 4 (comparison based on remission status).

Protein-wise ANOVA compared NPX-values between groups (day 0, day 3, day 10, week 4, week 24, and week 52) and results were adjusted for multiple testing using the Benjamini-Hochberg method. Significant differences were determined by *post-hoc* analysis (Tukey’s test).

Analyses were performed on Olink Insights Stat Analysis. Venn diagrams were created using the online tool InteractiVenn (15). Corresponding protein and gene names are listed in the **Supplementary Table 2**.

## Results

### Day 0 versus week 52: active disease versus remission

Compared to week 52 (stable remission for more than six months), 213 proteins were up-regulated, and 221 proteins were down-regulated at day 0 (active disease prior to administration of GC pulse therapy).

The ten most significant DEPs included: IL6R, PRTG, ITGAV (all three up-regulated), LBP, ITIH3, PLA2G2A, LRIG1, TNFSF14, TNC, and FURIN (all seven down-regulated, **Supplementary Figure 2**).

**Figure 1:**
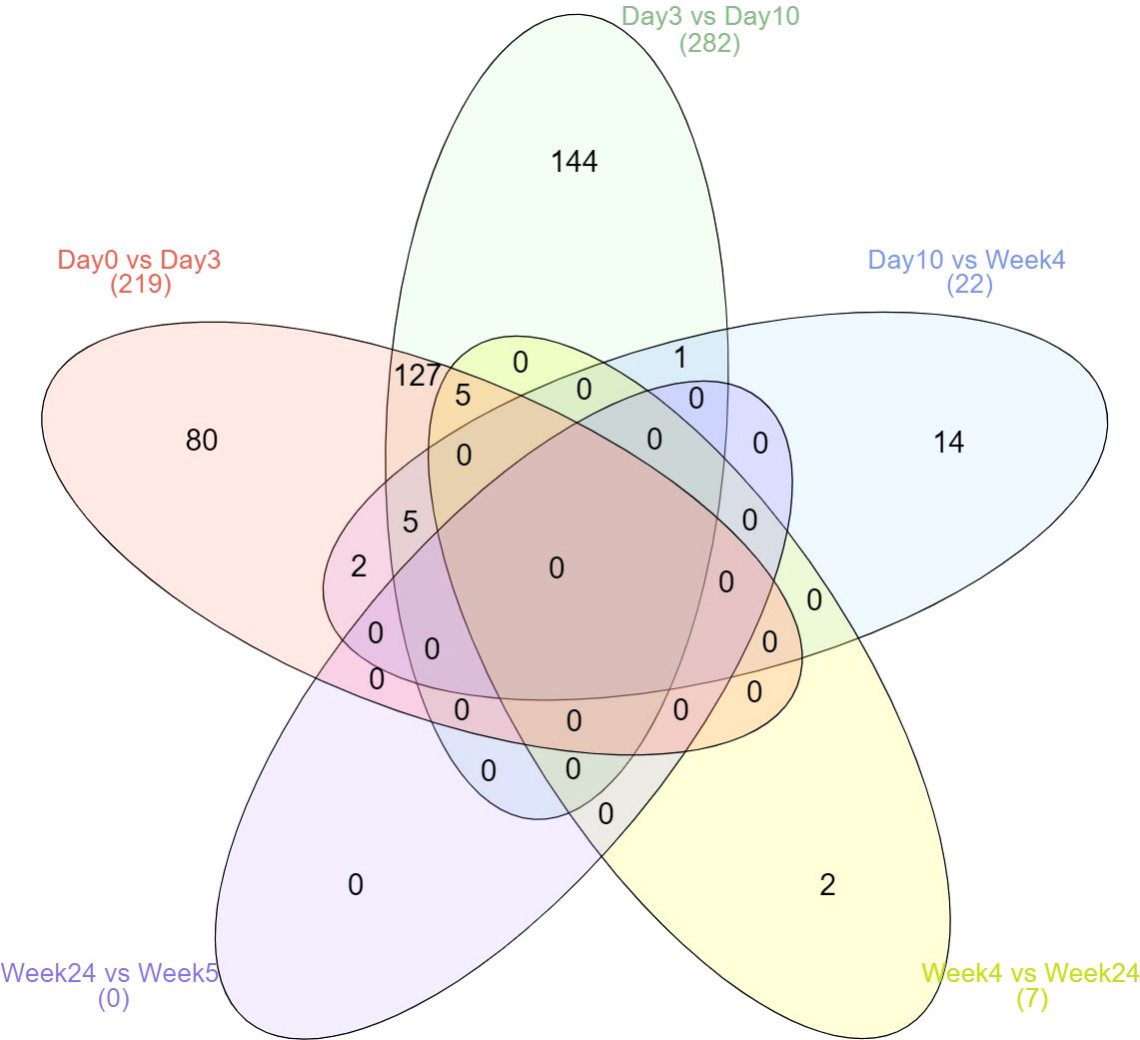
Kinetics of differential expressed proteins over the six time points. Displayed are the total number of differentially expressed proteins.

Most changes occurred within 10 days after treatment started (**Figure 1**). No difference was observed between weeks 24 and 52 during stable remission. No protein expression was continuously increased or decreased from day 3 to week 52. However, four proteins (CDON, IL6R, SIGLEC6, and ERBB2) were found continuously up- and two proteins (AREG and IL1R2) continuously down-regulated from day 3 to week 4. Two proteins (LBP, PLA2G2A) were continuously down-regulated from day 0 to day 10 and five proteins (IL6, MMP12, CXCL10, CCL7, CDCP1) were down-regulated from day 0 to day 3 and again from week 4 to week 24. One protein (IL6R) continuously increased from day 0 to week 4.

### Comparison of patients with and without prior GC treatment

Day 0 samples from patients with previous GC treatment (n=3) were compared to day 0 samples from patients without prior GC treatment (n=13). Only one protein, namely CLPP (down-regulated upon GC treatment), exhibited differential expression between the two groups (**Supplementary Figure 3**). As only one DEP was revealed, analysis in this study was performed for both groups combined.

In contrast, analysis of the two patients with > 1 day of prior oral GC treatment (3 and 7 days, respectively) as compared to patients without prior oral GC treatment revealed 36 DEPs. The ten most significant included MMP12, TNC, ANGPTL1, CHRDL1, GALNT3, CLPP, IL20RA, PREB (down-regulated upon GC treatment), and MMP3, CTSF (up-regulated upon GC treatment).

### Day 0 versus day 3: effect of pulsed GC treatment

**Figure 2:**
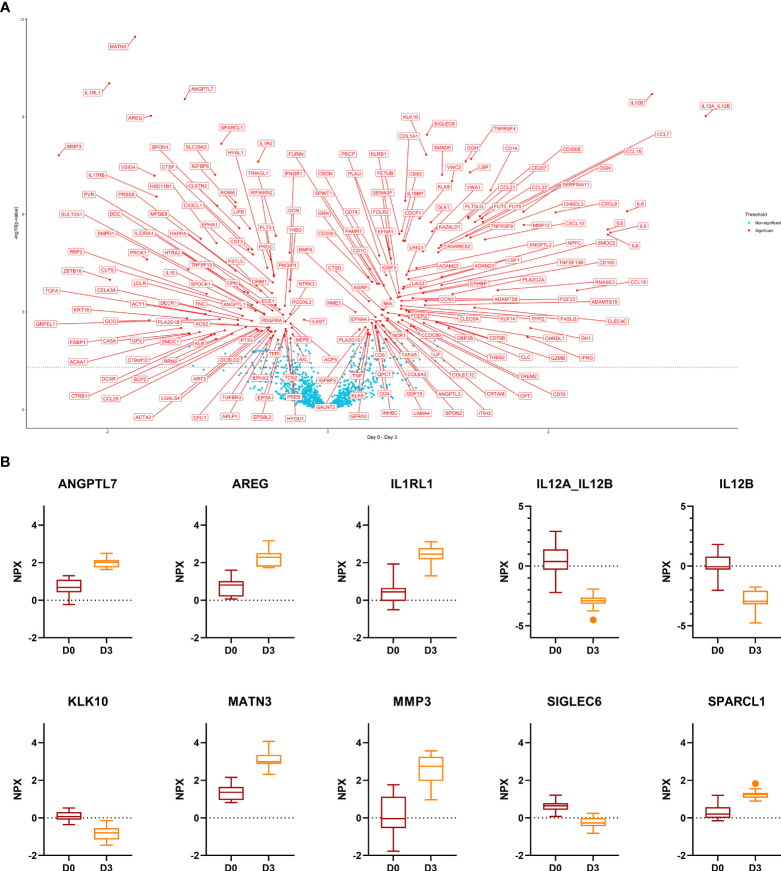
Comparison day 0 versus day 3. **(A)**, Volcano plot of the results from t-tests. The dots are colored based on the corresponding protein adjusted p-value. The dotted line represents a nominal p-value = 0.05. **(B)**, Boxplots of the 10 most significant differentially expressed proteins. Displayed are normalized protein expression (NPX) values. The box fill color indicates the sampling day. For all comparisons the significance is p < 0.0001.

In response to GC-pulse therapy for 3 days, the NPX-value of 208 proteins changed. In total, 94 proteins were up- and 114 down-regulated. The ten most significant DEPs included ANGPTL7, AREG, IL1RL1, MATN3, MMP3, SPARCL1 (up-regulated) and IL12A, IL12B, KLK10, SIGLEC6 (down-regulated) (**Figure 2**). Out of these ten proteins, only ANGPTL7 displayed the same trend upon remission (day 0 versus week 52). KLK10 displayed an opposite trend upon remission (increased levels at week 52 compared to day 0).

Twenty-five proteins were down- and 24 up-regulated with GC treatment and upon remission. On the other hand, we identified 19 DEPs that were down-regulated at day 3 and up-regulated at week 52 as compared to day 0, and 6 proteins (PROK1, TGFA, VSIG4, SMOC1, PTX3, TNC) that were up-regulated at day 3 and down-regulated at week 52 compared to day 0.

Upon GC-pulse therapy, many proteins showed impressive changes, which (almost) returned to pre-treatment levels, allowing an estimation of the kinetics and lasting effect of GC-pulse therapy. Further, we investigated the kinetics after day 3 of the 159/208 DEPs related to GC treatment that did not mirror disease activity to estimate the duration of the GC effect on serum proteins. We assessed the time to return to the day 0 levels for those proteins. 70/159 were up-regulated, and 89/159 were down-regulated upon day 3. After removal of those proteins (6 and 19, respectively) that displayed an opposite regulation upon GC treatment and remission, 64/70 proteins were up-, and 70/89 down-regulated on day 3. Most of those proteins returned to the day 0 level at day 10 (46/64 and 64/70). However, some proteins changed later, namely at week 4 (11/64 and 1/70), week 24 (4/64 and 4/70), and even at week 52 (3/64 and 1/70).

### Day 3 to week 4: weaning effect of GC and evolving effect of TCZ treatment

The interval between day 3 and week 4 reflects the weaning GC effect and the evolving TCZ effect. We identified a total of 497 DEPs between day 3 and week 4. The ten most significant DEPs included IL6R, CDON, IL12A, IL12B, CD83, TNFRSF4, ADAM23, IL6, FLT3LG, and SIGLEC6 (all up-regulated). IL6R, CDON, CD83, ADAM23, IL6, and FLT3LG displayed the same trend upon remission (higher level at week 52 as compared to day 0). IL12A, IL12B, TNFRSF4, and SIGLEC6 displayed no significant changes upon remission (week 52 as compared to day 0). In total, 303/497 proteins were up- and 194/497 down-regulated.

### Week 4 versus week 52: effect of TCZ

Based on the results above the changes measured between week 4 and week 52 are to an increasing extent induced by TCZ. Eleven DEPs (CDCP1, SOD2, CCL7, CXCL10, IL6, MMP12, CXCL9, CDON, TNFRSF8, TNC, and CLEC5A) were identified. All eleven DEPs were down-regulated at week 52 as compared to week 4. Five/11 DEPs displayed the same trend upon remission (CCL7, MMP12, CXCL9, TNC, CLEC5A). TNC, however, was up-regulated upon GC treatment (day 0 versus day 3), and CLEC5A displayed a slow kinetic – yet mirroring disease activity – with no changes between day 0 versus day 3, day 10, and week 4 but with down-regulation on day 0 versus week 24 and 52, respectively. On the other hand, 2/11 DEPs (IL6, CDON) displayed the opposite trend upon TCZ treatment (down-regulated) and remission (up-regulated).

Results of concomitant ultrasound analysis have been reported previously (16). There was an increasing wall thickness after day 3 on ultrasound as a possible expression of returning disease activity after stopping GC treatment. To investigate protein-expression that mirrors this disease activity-evolution, we identified DEPs with an opposite regulation upon remission, GC and TCZ treatment compared to day 3 versus week 4 or day 3 versus day 10. We identified 3 DEPs (CCL7, CXCL9, and MMP12).

At week 4 there was only one protein, IL4R, that was different between patients in remission (n=4) and patients not in remission (n=10). Patients in remission displayed lower levels of IL4R as compared to patients not in remission.

**Figure 3:**
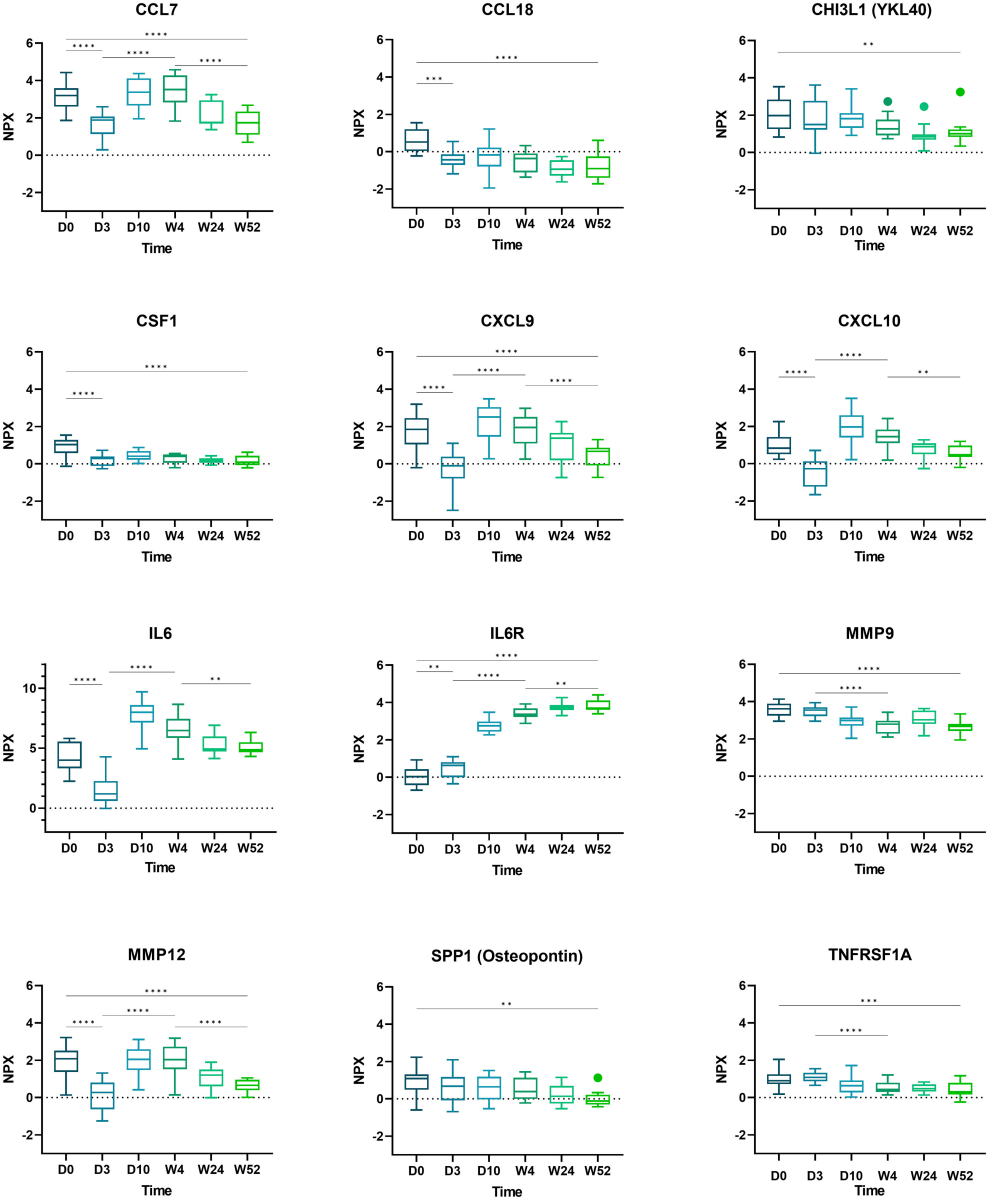
Boxplots of selected proteins from day 0 to week 52. Displayed are normalized protein expression (NPX) values. The box fill color indicates the sampling day. Displayed are significance levels for the comparison of day 0 versus day 3, day 0 versus week 52, day 3 versus week 4, and week 4 versus week 52. All significance levels are displayed in the **Supplementary Figure 4**. *p < 0.05, **p < 0.01, ***p < 0.001, ****p < 0.0001.1.

Boxplots of selected proteins of interest (CCL7, CCL18, CHI3L1, CSF1, CXCL9, CXCL10, IL6, IL6R, MMP9, MMP12, SPP1, and TNFRSF1A) are displayed in **Figure 3**.

## Discussion

Compared to patients in remission, newly diagnosed, active GCA patients showed 434/1463 differentially regulated proteins. 208/1463 proteins were regulated by pulsed GC treatment and 11/1463 by subsequent TCZ monotherapy. To our knowledge, this is the first study to report an in-depth analysis of serum proteins in new-onset GCA. The study design allowed for the comparative analysis of the effects of GC and TCZ treatment on the protein serum signature in GCA.

A three-day course of methylprednisolone resulted in the change of 14% of proteins assessed (208/1463, 14%). The high number reflects the combined genomic and non-genomic effects (17). In addition to remission-inducing, anti-inflammatory, and immune-suppressive effects, the data illustrate metabolic effects, demonstrating the central function of GC as a stress hormone. In line, we observed that most protein changes induced by GC treatment (159/208, 76%) were not congruent with remission. Similar data were recently reported in a transcriptome study of peripheral monocytes of GCA patients in remission (on < 10 mg/d prednisone for a minimum of 1 month *versus* off GC treatment): 3550 genes were differentially expressed in GC-treated patients (18). Remarkably, most of the 159/208 DEPs of our study had returned to initial levels at week 4. This finding argues for a limited duration of pulsed GCs, presumably between 10 days and 4 weeks.

Remission had a broader impact on serum protein expression in patients with GCA compared to pulse GC treatment and changed every third protein (434/1436, 30%). This profound impact of disease activity on serum proteomics in GCA is a novel finding. Longitudinally monitoring of protein expression revealed that these changes occurred early on. They were already seen within 10 days after initiation of treatment and concluded by week 24. Data published in 2018 on rheumatoid arthritis demonstrated a 24-week treatment with TCZ to result in broader proteomic changes toward healthy controls compared to infliximab or methotrexate. Of note, most of these changes occurred within 4 weeks (19). This is in line with our data depicting most changes within the same timeframe. In the GUSTO trial, 14/18 patients achieved clinical remission after a mean of 11.1 (8.3–13.9) weeks. This clinical profile is consistent with our proteomic data demonstrating that most changes in serum proteomics occurred during active disease, whereas no differences were observed during stable remission, i.e., after week 24. Median CRP levels were within the normal range from day 10 throughout the study to week 52, a known effect of TCZ treatment, and were therefore not helpful in assessing disease activity. Notably, a cross-sectional analysis conducted at week 4, which was based on remission status, revealed only one DEP as compared to the longitudinal analysis based on remission status. It is important to note that at week 4, the definition of remission was based solely on subjective findings such as symptoms. However, this approach remains a challenge in clinical practice as mild symptoms such as headaches are common and typically do not require treatment interventions. Additionally, this subjective definition of remission was not accompanied by significant differences at the protein level.

Based on the kinetics of GC-induced changes, any regulation of protein expression after week 4 is likely related to TCZ treatment. Between weeks 4 and 52, 11 DEPs were identified, and 5/11 (CCL7, MMP12, CXCL9, TNC, CLEC5A) proteins displayed the same trend upon TCZ treatment and during remission. Of these, TNC, associated with active AAV, was found to be up-regulated upon GC treatment and, therefore, might not be a reliable marker in conventional co-medication of GC plus TCZ (20). The serum levels of the four proteins CCL7, CXCL9, MMP12, and CLEC5A gradually declined towards remission, arguing for a potential role in monitoring GCA disease activity during TCZ-induced clinical remission and normalized CRP. Out of these, CLEC5A displayed a slow kinetic and might not be able to capture dynamic changes related to disease activity. Interestingly, these biomarkers cover different aspects of GCA pathobiology. CCL7 is involved in the recruitment of monocytes and has been identified previously as being overexpressed in monocytes of patients with active GCA (18). CXCL9 is linked to INFγ-related effects and B-cell trafficking in GCA, and up to now, TCZ treatment was not thought to interfere with INFγ-related signaling (21, 22). However, we observed decreased CXCL9 levels related to remission, GC, and importantly TCZ treatment. MMP12 participates in degrading elastic layers and basement membrane and is linked to GCA (23).

Due to their potential deleterious role in the destruction of the arterial vessel wall and formation of aneurysm, MMPs merit a special attention. In line with a previous study on RNA microarray analysis in temporal artery biopsies of GCA patients, we identified MMP12 and MMP9, which enable the trafficking of inflammatory cells to the vessel wall, as markers of disease activity in GCA (23). Of the eight MMPs assessed in our study (MMP1, 3, 7, 8, 9, 10, 12, and 13), four MMPs (MMP1, 8, 9, and 12) were down-regulated upon remission, and one out of these four (MMP12) upon GC treatment. MMP3 displayed no change upon remission but an increase upon GC treatment. In our phase-II RCT about TCZ to treat GCA, we reported high levels of MMP3 in clinical and serological full remission (6). Based on our current findings, the elevated levels of MMP3 in the RCT were most likely induced by the concomitant GC treatment and not as a reflection of disease activity. This is in line with another study in GCA that found MMP9 and MMP12 but not MMP3 levels to mirror disease activity (24). IL6 is known to induce the expression of the tissue inhibitor of metalloproteinases (TIMP) (25). Therefore, by blocking the IL6 pathway, TCZ blocks the natural inhibitor of tissue-destructive MMPs. The induction of MMP3 by GC, together with inhibition of TIMP by TCZ, explains the high risk of intestinal perforation but also the reported weakening of arterial wall with the development of aortal aneurysms. Taken together, the data strongly argue for a rapid reduction of concomitant GC.

As expected, there was an increase of the soluble IL6R related to TCZ treatment over time, achieving stable values after week 4. IL6, biologically not active under TCZ treatment, displayed peak levels at day 10 and a decline from day 10 to week 24, with stable values between week 24 and 52. This decline was not expected as IL6 levels did not decrease during TCZ treatment in the first RCT about TCZ to treat GCA (6). Another study demonstrated an early peak of IL6 levels approximately 14 days after initiation of TCZ with disease-specific differences (26). In a recent report, a decline of IL6 levels in 13/26 patients receiving TCZ treatment was associated with a low risk for early relapse (27). Collectively, the data are in line with the very low numbers of relapse in the GUSTO trial.

CSF1, a marker of active GCA that stimulates IL6 and GM-CSF production, decreased during the study (7, 28). Furthermore, we confirmed a significant decline of osteopontin (SPP1) (p=0.44) (6). Osteopontin has been proposed as a candidate to quantify disease activity in TCZ-treated patients and in chronic periaortitis (11, 29). Similar findings were reported in a study about biomarkers in systemic sclerosis (30). It showed a decrease in CCL18 and osteopontin levels under TCZ treatment. The authors postulated that IL6 and CSF1 stimulate monocytes to differentiate to macrophages secreting osteopontin and CCL18 (30). In GCA, a GM-CSF skewed MMP9^+^ macrophage subset produces CHI3L1, which may facilitate tissue destruction and angiogenesis (31). We observed decreased levels of CHI3L1 and MMP9 upon TCZ-treatment but no change upon GC-treatment. This is in line with prior findings indicating that CHI3L1 levels are elevated during active GCA and fail to normalize after GC-treatment (31).

In line with a previous study that assessed serum proteins of treatment-naive GCA patients as compared to healthy controls, we found elevated levels in active disease as compared to remission for TNC, TNFRSF1A, CSF1, MMP1, MMP9 and no change in LIF, MMP3, and IL33, IL1A protein levels (7).

A limitation of this study is the small number of patients. Furthermore, due to the statistical approach of a proof-of-concept trial with a Simon’s two stage design, there is no control population. Strengths are the homogeneous study population of new-onset GCA, most with histologically proven GCA, without any GC medication prior to study entry and the unbiased large protein profile.

One-third of the measured proteins covering wide biological functions changed in response to pulsed GC treatment, followed by TCZ monotherapy with most of the changes occurring within 10 days after treatment started. Several proteins were inversely induced by GC or TCZ. The data improve our understanding of the pathobiology of GCA and identify biomarkers, which detect subclinical disease activity beyond acute phase proteins such as C-reactive protein. They may qualify to monitor disease activity independent of treatment modality, e.g., IL6 blockade by TCZ. CCL7, CXCL9, and MMP12 should be further explored as biomarkers of disease activity in TCZ-treated GCA patients.

## Data availability statement

The original contributions presented in the study are publicly available. This data can be found here: https://osf.io/rqmtg/?view_only=c4cd2d9bf3b24b4283d9e62571930a8b.

## Ethics statement

The studies involving human participants were reviewed and approved by local ethics committee (Kantonale Ethikkommission Bern). The patients/participants provided their written informed consent to participate in this study.

## Author contributions

LC and PV initiated the study. AG, FB, FK, LC, PV, SR, and TG were involved in the study conception, design and implementation and data interpretation. AG, LC and PV drafted the manuscript. It was finalized by AG, FB, FK, LC, PV, SR, and TG. AG, LC, and PV had full access to all the data in the study, and accessed and verified the data. All authors contributed to the article and approved the submitted version.
