# Supplementary material for: Serum proteomics in giant cell arteritis in response to a three-day pulse of glucocorticoid followed by tocilizumab monotherapy (the GUSTO trial)
Source: Front Immunol. 2023 May 23;14:1165758. doi: 10.3389/fimmu.2023.1165758 (PMC10242646; doi:10.3389/fimmu.2023.1165758)
Supplement: Supplementary file 1 [file DataSheet_1.pdf]

frontiers

Supplementary Material

Serum proteomics in giant cell arteritis in response to a three-day pulse of glucocorticoid followed by tocilizumab monotherapy (the GUSTO trial)

Lisa Christ, MD[1*†], Andrea D. Gloor, MD[2†], Florian Kollert, MD[1], Timo Gaber, PhD[3], Frank Buttgereit, MD[3], Stephan Reichenbach, MD[1, 4], Peter M. Villiger, MD[5]

[1]Department of Rheumatology and Immunology, Inselspital, Bern University Hospital, University of Bern, Bern, Switzerland;
[2]Department of General Internal Medicine, Inselspital, Bern University Hospital, University of Bern, Bern, Switzerland;
[3]Department of Rheumatology and Clinical Immunology, Charité - Universitätsmedizin Berlin, Corporate Member of Freie Universität Berlin and Humboldt Universität zu Berlin, Berlin, Germany;
[4]University of Bern, Institute for Social and Preventive Medicine, Bern, Switzerland;
[5]Medical Center Monbijou, Rheumatology and Immunology, Bern, Switzerland

*** Correspondence:** Lisa Christ, MD, lisa.christ@insel.ch

† These authors contributed equally to this work and share first authorship

Supplementary figures

Supplementary Figure 1. Study treatment of the giant cell arteritis treatment with ultra-short glucocorticoids and tocilizumab (GUSTO) trial

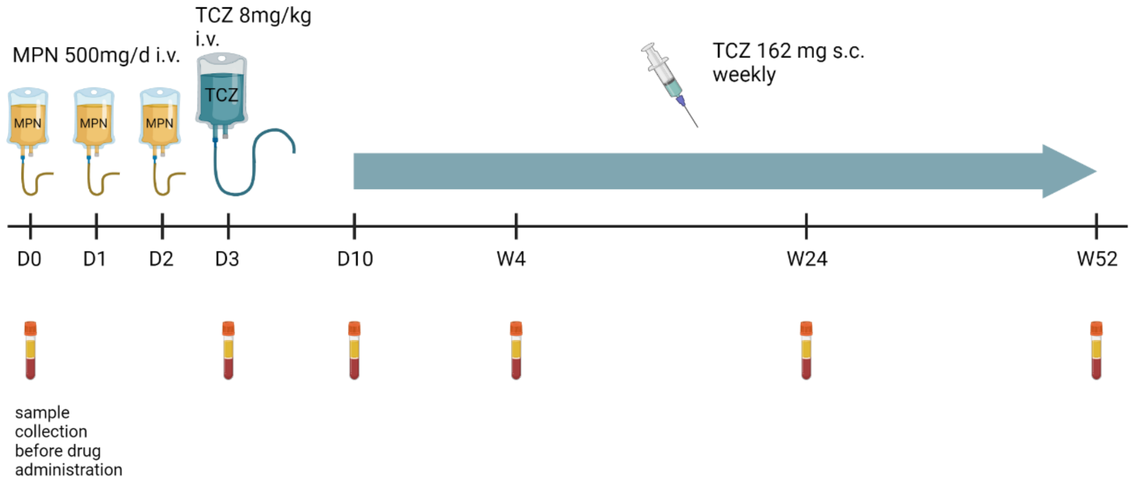

D=day. I.v.=intravenously. MPN=methylprednisolone. S.c.=subcutaneously. TCZ=tocilizumab. W=week.

Supplementary Figure 2. Boxplot of the 10 most significant differentially expressed proteins between day 0 and week 52

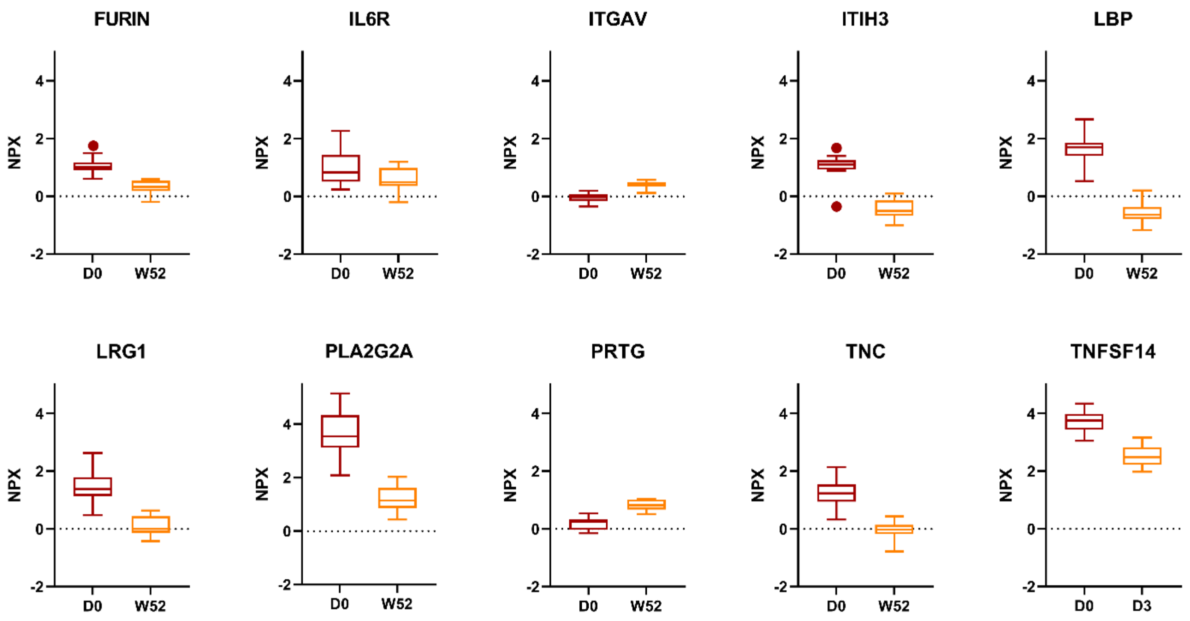

Displayed are normalized protein expression (NPX) values. The box fill color indicates the sampling day. For all comparisons the significance is $p < 0.0001$.

Supplementary Figure 3. Comparison of patients with and without prior glucocorticoid treatment

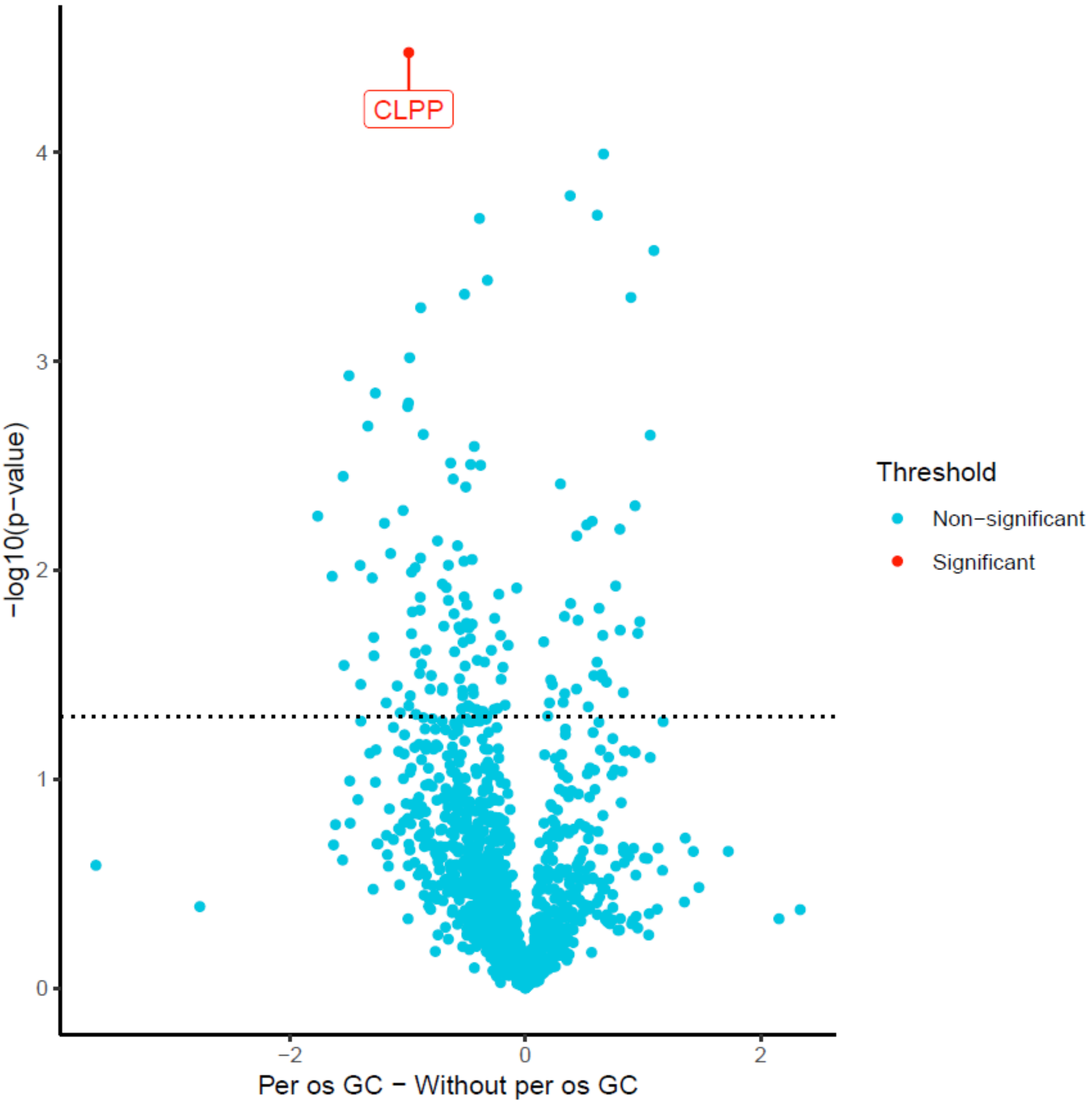

Volcano plot of the results from t-test. The signifcant assays are labeled with name. The dotted line represents the uncorrected significance threshold of 0.05. Only CLPP (down-regulated upon glucocorticoid treatment) was identified as differentially expressed between the two groups.

GC=glucocorticoid.

Supplementary Figure 4. Boxplots of selected proteins from day 0 to week 52

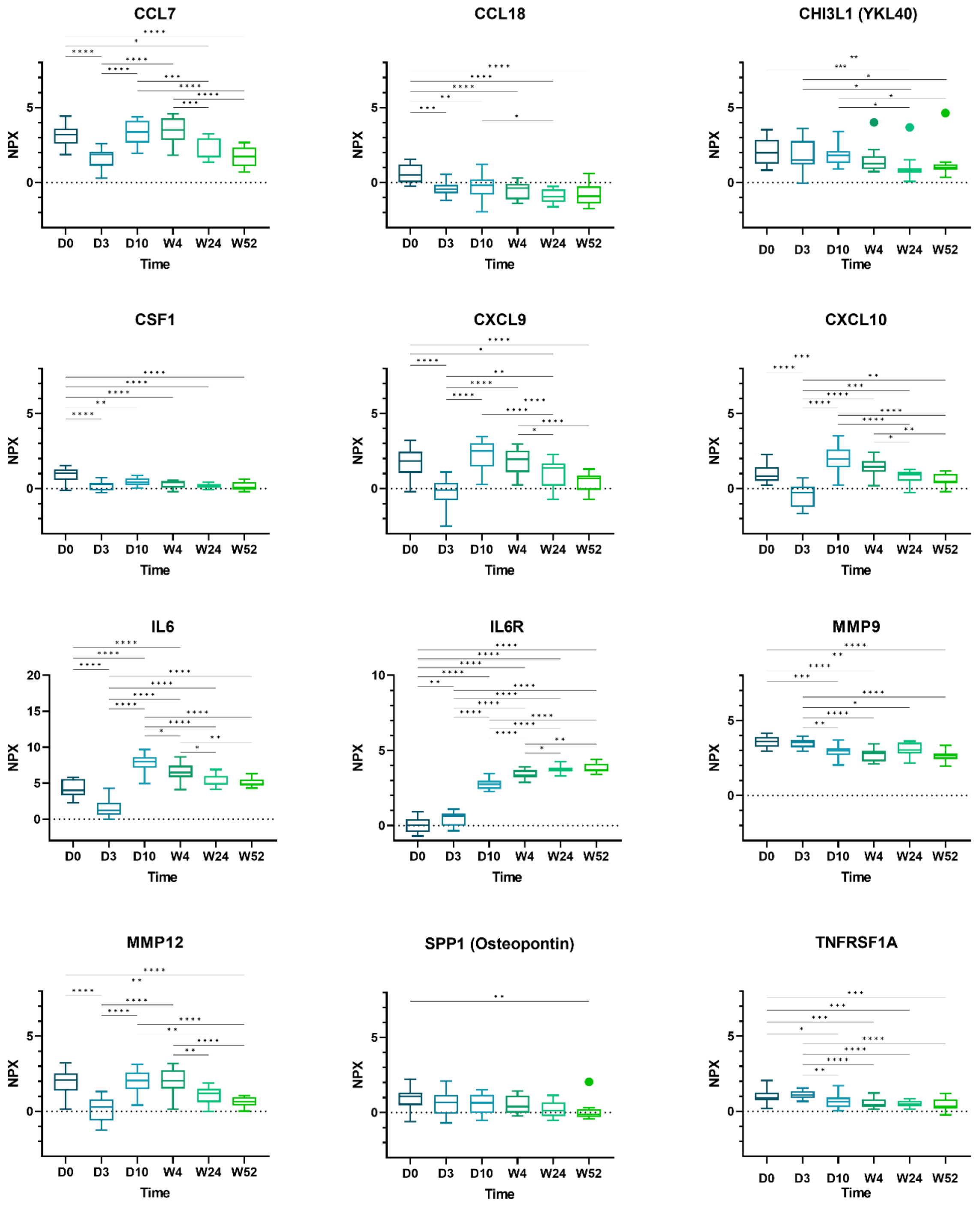

Displayed are normalized protein expression (NPX) values. The box fill color indicates the sampling day. *$p < 0.05$, **$p < 0.01$, ***$p < 0.001$, ****$p < 0.0001$.

Supplementary tables

Supplementary Table 1. Patients characteristics (N=16)

**Parameter**	**Values[1]**
Age [years]	72 (68-75)
Sex	
Female	11 (69%)
Male	5 (31%)
Ethnic origin	
White	16 (100%)
African-American	0 (0%)
Hispanic or Latino	0 (0%)
BMI [kg/m^2]	24 (23-26)
Prior GC treatment before baseline visit[2]	9 (56%)
Prior GC treatment before first serum sample collection[3]	3 (19%)
Days since GCA symptom onset	28 (18-58)
CRP at screening [mg/l]	61 (51-75)
CRP at baseline [mg/l]	44 (19-59)
ESR at screening [mm/h]	83 (61-88)
ESR at baseline [mm/h]	71 (50-79)
Cranial symptoms ever	14 (78%)
Headache	11 (69%)
Jaw claudication	10 (63%)
Visual symptoms	5 (31%)
PMR symptoms	9 (56%)
Weight loss >2kg/4 weeks	6 (38%)
Positive cranial ultrasound	14 (88%)
Aortitis on MRI	13 (81%)
Vasculitis on cranial MRI	13 (81%)

Positive histology	12 (75%)
Blood pressure at baseline visit [mmHg]	
Systolic right arm	135 (123-146)
Diastolic right arm	75 (68-81)
Systolic left arm	127 (120-143)
Diastolic left arm	75 (63-81)

[1]Values are n (%) or median (interquartile range). [2]For a median of 1 (1 - 4) days. [3]For a median of 1 (1, 3, and 7 days respectively) days.

BMI=body mass index. CRP=C-reactive protein. ESR=erythrocyte sedimentation rate. GC=glucocorticoids. GCA=giant cell arteritis. MRI=magnetic resonance imaging. PMR=polymyalgia rheumatica.

Supplementary Table 2. List of gene and corresponding protein names

Gene name	Protein name
ADAM23	Disintegrin and metalloproteinase domain-containing protein 23
ANGPT2	Angiopoietin-2
ANGPTL1	Angiopoietin-related protein 1
ANGPTL7	Angiopoietin-related protein 7
AREG	Amphiregulin
CCL7	C-C motif chemokine 7
CCL18	C-C motif chemokine 18
CCL19	C-C motif chemokine 19

CCL21	C-C motif chemokine 21
CD83	CD83 antigen
CD163	Scavenger receptor cysteine-rich type 1 protein M130
CDCP1	CUB domain-containing protein 1
CDON	Cell adhesion molecule-related/down-regulated by oncogenes
CHRDL1	Chordin-like protein 1
CHI3L1	Chitinase-3-like protein 1
CLEC5A	C-type lectin domain family 5 member A
CLPP	ATP-dependent Clp protease proteolytic subunit, mitochondrial
CTSF	Cathepsin F
CSF1	Macrophage colony-stimulating factor 1
CXCL9	C-X-C motif chemokine 9
CXCL10	C-X-C motif chemokine 10
CXCL13	C-X-C motif chemokine 13
ERBB2	Receptor tyrosine-protein kinase erbB-2
FLT3LG	Fms-related tyrosine kinase 3 ligand
FURIN	Furin
GALNT3	Polypeptide N-acetylgalactosaminyltransferase 3
KLK10	Kallikrein-10

IL1B	Interleukin-1 beta
IL2	Interleukin-2
IL1R2	Interleukin-1 receptor type 2
IL2RA	Interleukin-2 receptor subunit alpha
IL6	Interleukin-6
IL6R	Interleukin-6 receptor subunit alpha
IL12A	Interleukin-12 subunit alpha
IL12B	Interleukin-12 subunit beta
IL17	Interleukin-17
IL20RA	Interleukin-20 receptor subunit alpha
IL21	Interleukin-21
IL22	Interleukin-22
IL33	Interleukin-33
LIF	Leukemia inhibitory factor
LBP	Lipopolysaccharide-binding protein
ITGAV	Integrin alpha-V
ITIH3	Inter-alpha-trypsin inhibitor heavy chain H3
KLK10	Kallikrein-10
LBP	Lipopolysaccharide-binding protein

LRIG1	Leucine-rich repeats and immunoglobulin-like domains protein 1
MATN3	Matrilin-3
MMP1	Interstitial collagenase
MMP2	matrix metalloproteinase-2/gelatinase A
MMP3	Stromelysin-1
MMP8	Neutrophil collagenase
MMP9	Matrix metalloproteinase-9
MPP12	Macrophage metalloelastase
PDGF	Platelet-derived growth factor C
PLA2G2A	Phospholipase A2, membrane associated
PREB	Prolactin regulatory element-binding protein
PROK1	Prokineticin-1
PRTG	Protogenin
PTX3	Pentraxin-related protein PTX3
S100A9	calcium-binding protein A9
SIGLEC6	Sialic acid-binding Ig-like lectin 6
SMOC1	SPARC-related modular calcium-binding protein 1
SOD2	Superoxide dismutase [Mn], mitochondrial
SPARCL1	SPARC-like protein 1

SPP1	Osteopontin
TEK	Angiopoietin-1 receptor
TIMP1	Metalloproteinase inhibitor 1
TGFA	Protransforming growth factor alpha [Cleaved into: Transforming growth factor alpha
TNC	Tenascin
TNFRSF1A	Tumor necrosis factor receptor superfamily member 1A
TNFRSF4	Tumor necrosis factor receptor superfamily member 4
TNFRSF8	Tumor necrosis factor receptor superfamily member 8
TNFRSF14	Tumor necrosis factor receptor superfamily member 14
VEGF	Vascular endothelial growth factor
VEGFA	Vascular endothelial growth factor A
VSIG4	V-set and immunoglobulin domain-containing protein 4
